# Editorial: Tele-NeuroRehabilitation

**DOI:** 10.3389/fneur.2021.761690

**Published:** 2021-11-18

**Authors:** Annie J. Hill, Nam-Jong Paik, Swathi Kiran, Paolo Tonin

**Affiliations:** ^1^School of Health and Rehab Sciences, The University of Queensland and Surgical, Treatment and Rehabilitation Service, Metro North Health, Herston, QLD, Australia; ^2^Department of Rehabilitation Medicine, Seoul National University Bundang Hospital, Seongnam, South Korea; ^3^Speech, Language and Hearing Sciences, College of Health and Rehabilitation Sciences, Sargent College, Boston University, Boston, MA, United States; ^4^Department of Neurorehabilitation, Sant'Anna Institute, Crotone, Italy

**Keywords:** telerehabilitation or tele-rehabilitation, telemedicine, telehealth, home based therapy, e-health

In recent years, the role of telerehabilitation (TR) has gained increased importance across the continuum of the care process. It has been particularly relevant in the last 2 years after the explosion of a pandemic. However, some key points, such as the lack of common and shared methodologies for the delivery of TR treatments and the wide range of possible TR services (including telecounselling, telecare, telemonitoring, and teletherapy) have yet to be further analyzed. Therefore, there is a compelling need for studies to further investigate the effectiveness of many different clinical interventions in TR. The aim of this Research Topic is to present an update on the state of knowledge and expertise in different fields of TR for neurological patients. The papers described in this special issue span a range of neurological deficits, focussing on post-stroke impairments but also covering Parkinson's Disease, MCI, and pediatric neurological impairments. Likewise, while several studies describe preliminary/feasibility results, others report RCTs and comparisons with in-person control groups and conditions. Another theme in this Research Topic is the scoping review of the state of the art in TR in several parts of the world. We highlight those papers briefly here.

As highlighted in a recent Cochrane review, many studies have highlighted that a rehabilitative treatment produces equivalent results whether it is delivered in person or from a distance. While these findings are promising, this important concept cannot simply be generalized and needs to be analyzed in many different settings and patient populations to evaluate the feasibility of new TR approaches. In the article “A Feasibility Study of Expanded Home-Based Telerehabilitation After Stroke,” the authors evaluated the feasibility of several expansions, on treatment and assessment, to their prior randomized controlled TR study. The results of this study highlight the feasibility of adding new modules to a home-based system for patients with stroke and open the way to develop future holistic TR approaches, involving many aspects of treating patients at home, from intensive rehabilitation to the management of drugs.

Many articles of this Special Issue are focused on the treatment of aphasia via TR. In the article “Factors associated with adherence to self-managed aphasia therapy practice on a computer. A mixed-methods study alongside a randomized controlled trial,” the analysis focuses on factors that may impact on the adherence to self-managed computer-based aphasia treatment. Some factors determining greater or lesser adherence to remote asynchronous treatment emerged; particularly, it has been shown that patients were more willing to use computer therapy if they received supportive stimulation from a speech pathologist.

In the second article on aphasia, “Telerehabilitation for word retrieval deficits in bilinguals with aphasia: effectiveness and reliability as compared to in-person language therapy,” the authors verify whether a semantic treatment for word retrieval deficits in bilinguals with post-stroke aphasia yielded the same results if administered via TR or in person. Significant improvements were observed and the equivalence of treatment gains and adherence by participants between TR and in-person treatment was confirmed.

The goal of the article “The application of Lexical Retrieval Training in Tablet-Based Speech-Language Intervention” is to verify whether a widely used rehabilitation treatment (Lexical Retrieval Training) can be delivered remotely via a new application activated on a tablet. Initial observations showed that patients were partly able to use the tablet independently and needed to receive help from the operator; furthermore, the participants who received systematic training in the use of the tablet showed greater engagement with the therapy.

The feasibility and effectiveness of a new technological tool, designed for the clinical evaluation and remote treatment of aphasic disorders and cognitive deficits, are evaluated in the article “A virtual randomized control trial of a digital therapeutic for speech, language and cognitive intervention in post-stroke persons with aphasia.” The new software, delivered through a tablet, allows the clinician to remotely deliver the exercises in an asynchronous mode, along with a protocol individualized for each patient to improve their adherence to the study. The results highlighted the feasibility and tolerability of remote TR treatment; furthermore, patients in the experimental group showed greater clinical improvements than the control group.

A TR treatment for aphasia delivered 6 years after the onset of aphasia is unusual. Such treatment is described in the article “Integrated Discourse Therapy After Glioblastoma: A Case Report of Face-to-Face and Tele-NeuroRehabilitation (TNR) Treatment Delivery.” Although the participant had long-term language disabilities, integrated conversation therapy was administered face-to-face and remotely via TNR. The results showed improvements after both types of treatment administration. Additionally, a satisfaction survey indicated a preference for delivering TNR treatment.

Studies on TR for patients with cognitive disorders are not yet well-defined. The article “Analysis of feasibility, adherence, and appreciation of a newly developed telerehabilitation program for people with Mild Cognitive Impairment (MCI) or Vascular Cognitive Impairment (VCI)” addresses this problem, verifying the feasibility, adherence, and acceptance of a new TR system that provides patients with MCI a multidimensional program to prevent further cognitive decline. The results of this preliminary study showed a good feasibility of the new TR system, and a positive level of adherence to the program and satisfaction with the technological approach.

TR in children requires a very specific approach and careful consideration in terms of delivery. Two papers in this Special Issue address this topic. The article “Adaptive Working Memory Training Can Improve Executive Functioning and Visuo-Spatial Skills in Children with Pre-term Spastic Diplegia (pSD)” shows the results of a study aimed at improving executive function and visuo-spatial skills with evidence-based training focused on working memory (WM) in children with pSD. The treatment was remotely delivered to the patients' homes. The results showed an improvement in targeted WM abilities and generalization to other neuropsychological functions, such as visuo-spatial processing, inhibition, and phonological processing.

The article “Feasibility Analysis of CareToy-Revised Early Intervention in Infants at High Risk for Cerebral Palsy (CP)” evaluates a new technological system (CareToy-CT) that provides via TR a highly personalized, family-centered, home-based early intervention for young infants. CT, already used with pre-teens without brain injury, has been adapted for CP high-risk infants. The preliminary reports evidenced the feasibility of this innovative early intervention in infants at high risk for CP.

The feasibility of a new TR technological system is evaluated in the article “Exergaming as part of telerehabilitation can be adapted to outpatient training: preliminary results of a non-randomized pilot study in Parkinson's disease.” The authors built a TR exergaming system, with virtual pick & place tasks for small hand movements, that was designed specifically for PD patients. The preliminary results of a pilot study confirmed that this home exergame, delivered by TR support, can provide clinical efficacy comparable to that of hospital training. In addition, the exergame looks motivating and easy to use.

The innovative aim of assessing whether teleassement is feasible in post-stroke patients is assessed in the article “Remote Assessment of Post-Stroke Elbow Function Using Internet-Based Telerobotics: A Proof-of-Concept Study.” The authors created a system with a master robot interacting with a doctor and a slave robot interacting with the elbow of a subject with a stroke, linked together by the Internet. To test the feasibility of remote assessment in an extreme scenario, the teleassessment examiner was in Bethesda (USA) and stroke patients were in Seoul (South Korea). The results of this pilot study showed that this innovative telerobotic evaluation is feasible even in the worst situation.

A relevant trend in TR is the implementation of multidimensional rehabilitation systems, to make the continuity of care more complete. This multidimensional concept emerges in the article “Virtual reality for motor and cognitive rehabilitation from clinic to home: A pilot feasibility and efficacy study for persons with chronic stroke.” The feasibility of an innovative digital system to deliver together motor and cognitive rehabilitation treatments is compared in the clinical context and at home. The results showed that the protocol is feasible and the satisfaction and adherence to the treatment were good in both conditions.

From the perspective of TR as a multidimensional system, the social networks in which post-stroke patients live are relevant. The article “Social network structure is related to functionalimprovement from home-based telerehabilitation after stroke” addresses this aspect. A pilot group of post-stroke patients underwent periodic assessments for motor function and mood and remote supervision through a TR system. The social network of TR patients was positively correlated with the improvement of the clinical picture and appeared wider than that of the control group, highlighting that the strengthening of the social network could be crucial to improving the effects of TR.

A TR approach for people in the vegetative state (VS) or minimally conscious state (MCS) is uncommon. The article “Telemonitoring of patients with chronic traumatic brain injury: a pilot study” evaluates a new specific telemonitoring program, based on an advanced video-conferencing system and wearable monitoring devices. A comparison between a group of patients monitored at home through the new TR program and a group hospitalized in a traditional long standing hospital showed a trend toward a better clinical picture and a lower daily cost of care in patients treated at home.

The difficulties of patients with chronic neurological disabilities and their caregivers who attend clinical visits in specialized centers are analyzed in the article “The Time Burden of Specialty Clinic Visits in Persons with Neurologic Disease: A Case for Universal Telemedicine Coverage.” A specific survey showed that many patients and caregivers experienced severe problems, transportation difficulties, travel times, and daily schedule changes. The results of this survey underscore the potential role of TR in minimizing this burden.

Balance instability is a frequent and severely disabling symptom in Parkinson's patients. On the other hand, many studies have shown that Tai Chi is an aerobic exercise that improves the ability to balance and prevents falls in elderly people. These observations lead to a hypothesis that a mobile phone application combined with a Tai Chi intervention may be effective in improving the balance ability of patients with PD. To verify this hypothesis, the article “A mobile phone App-based Tai Chi training in Parkinson's disease (PD): protocol for a randomized control study” describes the protocol for a proposed single-blind RCT.

Despite its growing body of literature and the scope of services in more developed countries, telerehabilitation continues to face challenges and obstacles to its emergence in less developed countries. Three articles in this special issue focus on the situation of TR in different countries.

In the article “Challenges to the Emergence of Telerehabilitation in a Developing Country: A Systematic Review” the authors focused on the situation in the Philippines. This review was based on an extensive literary search, including gray literature. The overall conclusion is a paucity of data on TR in the Philippines. An alarming detail is that the most common barriers (slow internet speeds, legal concerns, and skepticism) involve all three key factors necessary for the realization of a TR service: technical, organizational, and human.

The article “Telemedicine Guidelines in South East Asia (SEA)—A Scoping Review” aims to explore and compare guidelines on telehealth and telemedicine in South East Asian countries. After a very large search, it emerged that only five countries contained some form of guidelines on telemedicine; the National Telemedicine Guidelines of Singapore are the most comprehensive guidelines in the SEA region and the only one comparable with other telemedicine guidelines worldwide.

The article “Tele-Neuro-Rehabilitation in Italy: state of the art and future perspectives” evaluates the role of telerehabilitation in Italy, as regards motor and cognitive rehabilitation programs. The analysis of this review shows that the studies on TR achieved satisfactory results, however, the quality of the research needs to be significantly improved to clarify the benefits and risks of remote care. Furthermore, the samples from these studies are small, making the results of little relevance to a substantial economic analysis.

In the article “Using the technology acceptance model to identify factors that predict the likelihood of adopting tele-neurorehabilitation,” some predictors that could explain why teleneurorehabilitation has not yet reached mainstream adoption, are discussed. The data analysis suggests that poor computer self-efficacy, high computer anxiety, low perception of usefulness, and a belief that the technology is not user-friendly are significant predictors of an individual's likelihood to use TR. The authors underline that if TR moves too soon from the domain of research to become the core business of the health sector, key stakeholders will face barriers that have consistently hindered adoption.

The editors hope that this collection of articles sheds some light on the barriers and facilitators to the advancement of telerehabilitation in neurological patient populations. The innovative research being done in this sphere will no doubt lead to better outcomes for our patients and improved access to the services they need, even in pandemic circumstances.

## Author Contributions

PT, AH, N-JP, and SK participated in the same way in the editing of the text. All authors contributed to the article and approved the submitted version.

## Conflict of Interest

The authors declare that the research was conducted in the absence of any commercial or financial relationships that could be construed as a potential conflict of interest. The handling editor declared a past co-authorship with author PT.

## Publisher's Note

All claims expressed in this article are solely those of the authors and do not necessarily represent those of their affiliated organizations, or those of the publisher, the editors and the reviewers. Any product that may be evaluated in this article, or claim that may be made by its manufacturer, is not guaranteed or endorsed by the publisher.

